# In vitro synergy between sodium deoxycholate and furazolidone against enterobacteria

**DOI:** 10.1186/s12866-019-1668-3

**Published:** 2020-01-06

**Authors:** Vuong Van Hung Le, Catrina Olivera, Julian Spagnuolo, Ieuan G. Davies, Jasna Rakonjac

**Affiliations:** 1 School of Fundamental Sciences, Massey University, Palmerston North, New Zealand; 2 Present Address: Department of Biomedicine, University Hospital Basel, 4031 Basel, Switzerland; 3 New Zealand Pharmaceuticals Ltd, Palmerston North, New Zealand

**Keywords:** Furazolidone, Nitrofurans, Sodium Deoxycholate, Antimicrobial combination, Synergy, Enterobacteria

## Abstract

**Background:**

Antimicrobial combinations have been proven as a promising approach in the confrontation with multi-drug resistant bacterial pathogens. In the present study, we identify and characterize a synergistic interaction of broad-spectrum nitroreductase-activated prodrugs 5-nitrofurans, with a secondary bile salt, sodium deoxycholate (DOC) in growth inhibition and killing of enterobacteria.

**Results:**

Using checkerboard assay, we show that combination of nitrofuran furazolidone (FZ) and DOC generates a profound synergistic effect on growth inhibition in several enterobacterial species including *Escherichia coli*, *Salmonella enterica*, *Citrobacter gillenii* and *Klebsiella pneumoniae*. The Fractional Inhibitory Concentration Index (FICI) for DOC-FZ synergy ranges from 0.125 to 0.35 that remains unchanged in an ampicillin-resistant *E. coli* strain containing a β-lactamase-producing plasmid. Findings from the time-kill assay further highlight the synergy with respect to bacterial killing in *E. coli* and *Salmonella*.

We further characterize the mechanism of synergy in *E. coli* K12, showing that disruption of the *tolC* or *acrA* genes that encode components of multidrug efflux pumps causes, respectively, a complete or partial loss, of the DOC-FZ synergy. This finding indicates the key role of TolC-associated efflux pumps in the DOC-FZ synergy. Overexpression of nitric oxide-detoxifying enzyme Hmp results in a three-fold increase in FICI for DOC-FZ interaction, suggesting a role of nitric oxide in the synergy. We further demonstrate that DOC-FZ synergy is largely independent of NfsA and NfsB, the two major activation enzymes of the nitrofuran prodrugs.

**Conclusions:**

This study is to our knowledge the first report of nitrofuran-deoxycholate synergy against Gram-negative bacteria, offering potential applications in antimicrobial therapeutics. The mechanism of DOC-FZ synergy involves FZ-mediated inhibition of TolC-associated efflux pumps that normally remove DOC from bacterial cells. One possible route contributing to that effect is via FZ-mediated nitric oxide production.

## Background

Antimicrobial resistance (AMR) is one of the most serious threats with which humans have been confronted. A UK-Prime-Minister-commissioned report in 2014 estimated that AMR, without appropriate interventions, will cause globally 10 million deaths per annum with a cumulative loss of US $100 trillion by 2050 [1]. In this dire context, alternative approaches are urgently needed besides the discovery of novel antibiotics. Antimicrobial combinations have been proven to be a promising approach with some widely accepted advantages, including enhancement of antimicrobial efficacy, deceleration of resistance development rate and alleviation of side effects by lowering the doses of two drugs [2, 3]. Moreover, this approach could amplify the significance of ongoing antimicrobial discovery programs; particularly the advent of any novel antimicrobial compound would bring about a large number of possible double combinations with existing antimicrobial agents to be evaluated, let alone triple and quadruple combinations.

Sodium deoxycholate (DOC) (Additional file 1: Figure S1e) is a facial amphipathic compound in bile, which is secreted into the duodenum to aid lipid digestion and confer some antimicrobial protection [4]. Though extensive research has been conducted to elucidate the interaction between DOC, either alone or in the bile mixture, and enteric bacteria, the mode of its antimicrobial action remains elusive. It was suggested that DOC could attack multiple cellular targets, including disturbing cell membranes, causing DNA damage, triggering oxidative stress and inducing protein misfolding [4–6]. Nonetheless, Gram-negative bacteria such as *Escherichia coli* and *Salmonella* are highly resistant to DOC by many mechanisms such as employment of diverse active efflux pumps, down-regulation of outer membrane porins and activation of various stress responses [5, 7–9].

The 5-nitrofurans are an old class of synthetic antimicrobials, clinically introduced in the 1940s and 1950s [10]; several are commercially available, including furazolidone (FZ), nitrofurantoin (NIT) and nitrofurazone (NFZ) (Additional file 1: Figure S1). FZ is used to treat bacterial diarrhea, giardiasis and as a component in combinatorial therapy for *Helicobacter pylori* infections; NIT and NFZ are used for urinary tract infections and topical applications, respectively [11]. They are prodrugs which require reductive activation, which is mediated in *E. coli* largely by two type-I oxygen-insensitive nitroreductases, NfsA and NfsB, and in their absence by type II oxygen-sensitive nitroreductase, AhpF [12]. NfsA and NfsB perform stepwise 2-electron reduction of the nitro moiety of the compound into two redox-reactive nitroso and hydroxylamino intermediates and biologically inactive amino-substituted product [13, 14]. Detailed mechanism of how bacterial cells are killed by the reactive intermediate(s) is yet to be clarified. Nevertheless, it has been proposed that the hydroxylamino derivatives could trigger DNA lesions, disrupt protein structure and arrest RNA and protein biosynthesis [15–18]. Some reports also suggested that nitric oxide could be generated during the activation process, thus inhibiting the electron transport chain of bacterial cells although clear evidence for that is not available so far [19, 20]. It is worth mentioning that nitroreductase-encoding genes are not only commonly present in enterobacteria but also found in other bacterial species such as *Staphylococcus aureus*, *Bacillus subtilis*, *Vibrio fischeri* and parasites (e.g. *Trypanosoma brucei*, *Leishmania major*) [21–24]. Nitroreductase enzymes play different physiological roles in different species; in *E. coli,* multiple functions have been proposed for NfsA and NfsB, including dihydropteridine reductase, chromate reductase, quinone-dependent azo reductase, and part of the oxidative stress response [21].

In this study, we have characterized the interaction of DOC with FZ and other three related nitrofurans against a range of enterobacteria. We identified the underlying mechanism of DOC-FZ synergy using *E. coli* K12 as a model organism.

## Results

### The synergy between DOC and 5-nitrofurans against enterobacteria

**Fig. 1:**
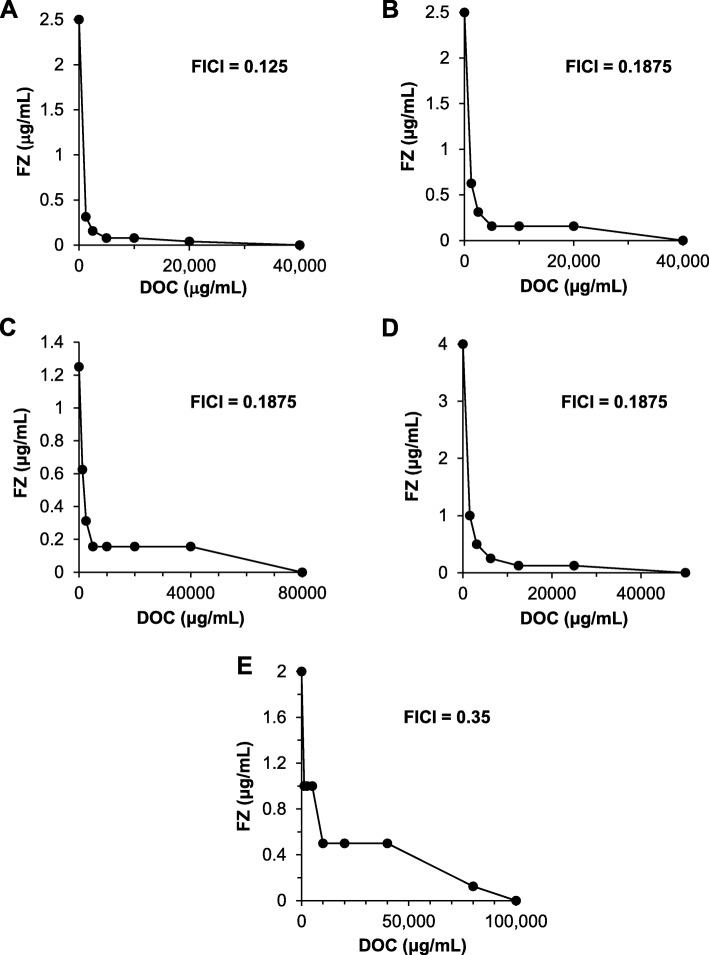
FZ interaction with DOC in growth inhibition of streptomycin- resistant *E. coli* K12 (**a**), ampicillin- and streptomycin-resistant *E. coli* K12 (**b**), *Salmonella enterica* subsp. *enterica* serovar Typhimurium LT2 (**c**), *Citrobacter gillenii* (**d**) and *Klebsiella pneumoniae* (**e**). Graphs (isobolograms) were obtained using a checkerboard analysis at multiple concentrations of each molecule. Each data point represents the minimum molecule concentrations alone or in combination causing 90% inhibition of bacterial growth

To evaluate the synergy between DOC and FZ, the checkerboard growth inhibition assays were performed for several enterobacteria, including *Salmonella enterica* subsp. *enterica* serovar Typhimurium LT2, *Citrobacter gillenii, Klebsiella pneumoniae* and two *E. coli* antibiotic-resistant laboratory strains (streptomycin-resistant and streptomycin/ampicillin-resistant). DOC and FZ act synergistically in inhibiting growth of the microorganisms listed (Fig. 1), with FICI ranging from 0.125 in streptomycin-resistant *E. coli* strain (Fig. 1a) to 0.35 in *K. pneumoniae* (Fig. 1e). DOC-FZ synergy was also observed against two *E. coli* pathogenic strains (*E. coli* strain O157 and urinary tract infection strain P50; Additional file 1: Figure S2). It is worth noting that, when used alone, very high DOC concentrations were required to exert an equivalent effect on inhibiting the growth of these Gram-negative enterobacteria, whereas the concentration in combination with FZ at the lowest FICI was within the range of the bile salts concentration in the human intestine (2.5 mg/mL or 6 mM) [25].

We also examined the interaction between DOC and other nitrofuran compounds, including NIT, NFZ and CM4 (a 5-nitrofuran compound we discovered during an antimicrobial screening campaign against *E. coli*, Additional file 1: Figure S1d) in all bacterial species mentioned above. We found that NIT, NFZ and CM4 were synergistic with DOC in *E. coli* laboratory strain (Fig. 3), *Citrobacter gillenii* (Additional file 1: Figure S3) and *Salmonella enterica* Typhimurium LT2 (Additional file 1: Figure S4). By contrast, the interaction between NIT or NFZ and DOC was indifferent in *K. pneumoniae* isolate (Additional file 1: Figure S5). CM4 did not inhibit growth of this *Klebsiella* strain in the range of concentrations used in the experiment (up to 256 μg/ml), hence the interaction could not be defined.

**Fig. 2:**
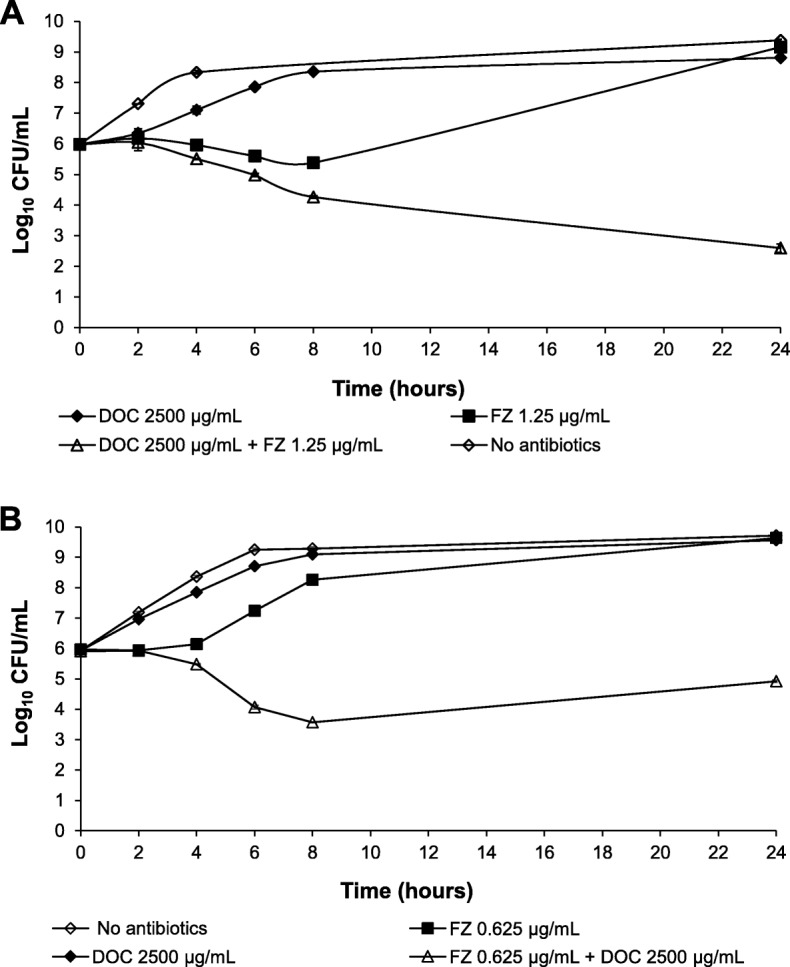
Time- kill analysis of the DOC and FZ combination in killing *E. coli* strain K1508 (**a**) and *Salmonella enterica* serovar Typhimurium LT2 (**b**). The data is presented as the mean ± standard error of the mean (SEM) of three independent measurements. The count of the live cells was determined at indicated time points by titration of colony-forming units on agar plates. The lower limit of detection was 60 CFU/mL

To investigate the interaction between DOC and FZ in terms of bactericidal effects, the time-kill assay was employed. Streptomycin-resistant *E. coli* K12 laboratory strain K1508 and *S. enterica* serovar Typhimurium strain LT2 were exposed to sub-inhibitory concentrations of DOC (2500 μg/mL) alone, or FZ (0.5 × MIC) alone, or combination of the two drugs at such sub-inhibitory concentrations, over a 24 h period. The sample was taken at different time points and the surviving bacteria were titrated on the antimicrobial-free plates. Centrifugation and resuspension were applied for each sample to eliminate antimicrobial carryover before plating. After 24 h, the total cell count in the sample treated with the DOC-FZ combination was about five to six orders of magnitude lower than that in the sample treated with either DOC or FZ alone for both *E. coli* and *Salmonella* (Fig. 2), demonstrating the synergy in bacterial killing between DOC and FZ.

### The role of AcrAB-TolC efflux pump in synergistic interaction between DOC and nitrofurans

One commonly accepted principle is that the synergy between two drugs is a consequence of one drug suppressing bacterial physiological pathways that mediate resistance to the other one. It has been reported that DOC could be expelled out of the cell via a wide range of efflux pumps, in which the tripartite efflux system AcrAB-TolC plays the major role [7, 8]. This led us to hypothesize that FZ may inhibit the activity of efflux pumps, thus allowing intracellular accumulation of DOC to exert its lethal effect. If this scenario were true, disruption of the efflux pumps’ function by mutation was expected to make this activity of FZ redundant, thus increasing the interaction index (FICI) in the mutant strains.

**Fig. 3:**
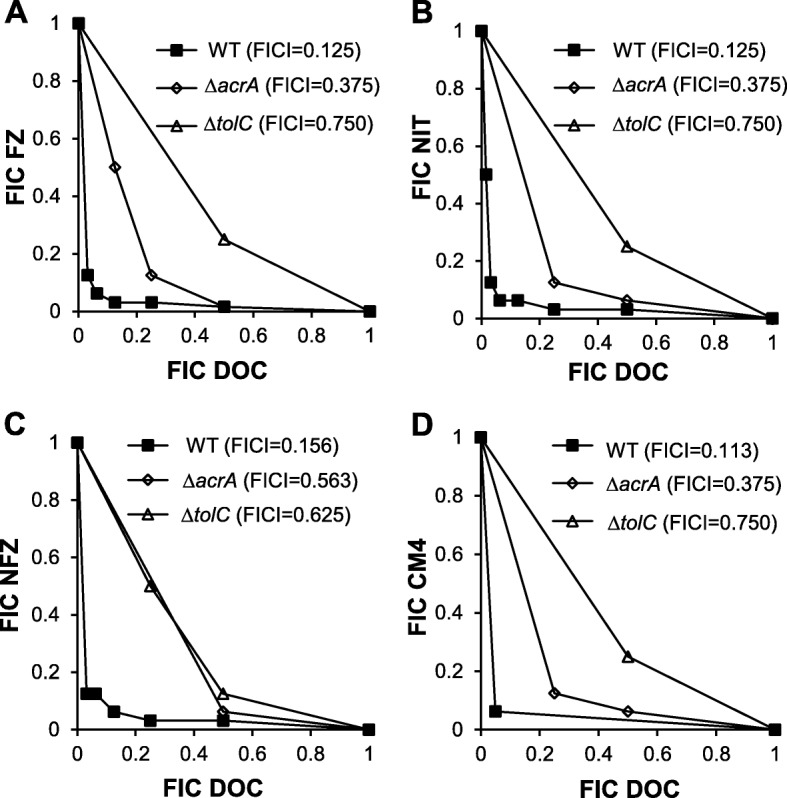
Effect of the Δ*tolC* and Δ*acrA* mutations on DOC synergy with FZ, NIT, NFZ and CM4 in *E. coli*. Isobolograms characterizing interactions of DOC with FZ (**a**), NIT (**b**), NFZ (**c**) and CM4 (**d**) in growth inhibition assays of the *E. coli* K12 strain K1508 (WT or wild-type and two isogenic deletion mutants, Δ*acrA* and Δ*tolC*). Each data point corresponds to the FIC (ratios of the 90% growth inhibition concentrations in combination *vs.* alone) for one of the four nitrofurans (y axis) and DOC (x axis). The Δ*tolC* strain (K2403) had the MICs for FZ, NIT, NFZ, CM4 at 1.25 μg/mL, 4 μg/mL, 8 μg/mL and 4 μg/mL, respectively. The Δ*acrA* strain (K2424) had the MICs for FZ, NIT, NFZ, CM4 at 2.5 μg/ml, 8 μg/mL, 8 μg/mL and 8 μg/mL, respectively. The WT strain K1508 had the MICs for FZ, NIT, NFZ, CM4 at 2.5, 32, 16 and 32 μg/mL, respectively

To validate this model in *E. coli*, checkerboard assays were performed on the strains containing deletions of individual genes encoding the AcrAB-TolC efflux pump system, Δ*tolC* and Δ*acrA*. Deletion of *tolC* caused a shift from the synergistic interaction between DOC and FZ in the wild type (FICI = 0.125) to indifferent interaction (FICI = 0.75; Fig. 3a). The Δ*acrA* mutant exhibited a 3-fold increase in the FICI relative to the isogenic wild type strain. Such changes were also observed for the interaction between DOC and other nitrofurans, NIT, NFZ or CM4 (Fig. 3b, c and d).

**Fig. 4:**
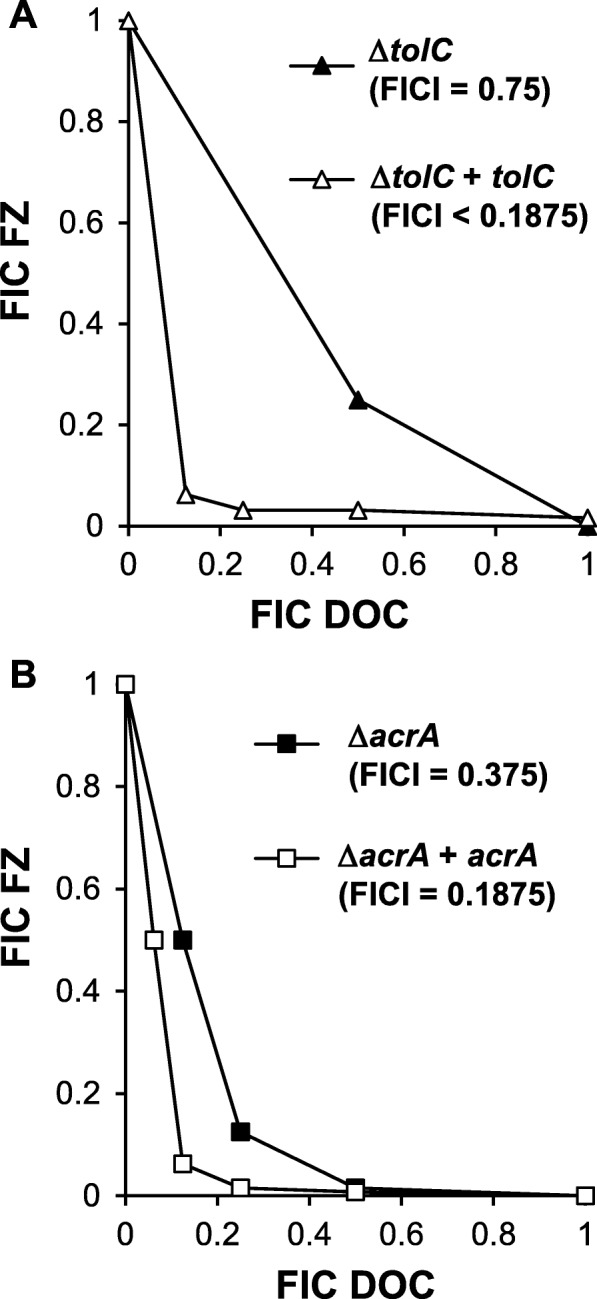
Recovery of FZ-DOC synergy in complemented Δ*tolC* and Δ*acrA* mutants. Isobolograms of FZ-DOC interactions in growth inhibition of: **a** Δ*tolC* mutant (Δ*tolC*) and a derived strain containing a plasmid expressing *tolC* gene (Δ*tolC* + *tolC*); **b** Δ*acrA* mutant (Δ*acrA*) and a derived strain containing a plasmid expressing *acrA* gene and (Δ*acrA* + *acrA*). Each data point corresponds to the FIC (ratios of the 90% growth inhibition concentrations in combination *vs.* alone) for FZ (y axis) and DOC (x axis)

To confirm that these observations were conferred by direct effect of the *tolC* and *acrA* deletion, rather than indirect effects of other genes or proteins, complementation of the corresponding deletion mutations by plasmid-encoded *tolC* and *acrA* was performed. To compensate for the multiple copies of plasmid-containing genes, complementation was carried out at a low level of expression, nevertheless it completely restored the strong synergy between DOC and FZ in these complemented strains (Fig. 4). These findings collectively support the model that the efflux pumps act as the interacting point for the synergy between DOC and FZ.

**Fig. 5:**
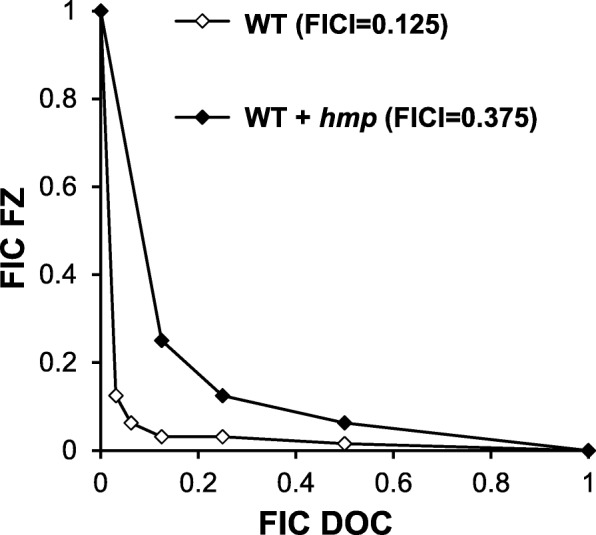
Effect of the *hmp* gene overexpression on FZ-DOC synergy. The isobologram of DOC and FZ interaction in *E. coli* having differential expression of NO-detoxifying protein Hmp. WT, *E. coli* laboratory strain K1508; WT + *hmp*, K1508 containing a plasmid expressing Hmp under the control of a T5-lac hybrid promoter. Expression of *hmp* gene was induced by IPTG (1 mM). Each data point corresponds to the FIC (ratios of the 90% growth inhibition concentrations in combination *vs.* alone) for FZ (y axis) and DOC (x axis)

An intriguing question to be unraveled is how FZ could negatively influence the action of efflux pumps. We hypothesized that FZ could lower the energy supply to efflux pumps by mediating an increase in concentration of nitric oxide (NO). To verify the proposed model, the interaction between DOC and FZ in an *E. coli* strain with increased expression of protein Hmp (the *E. coli* nitric oxide dioxygenase) was inspected. The rationale for this is that overexpression of Hmp protein would increase detoxification of NO by conversion into benign NO_3_^–^ ions, thus relieving the effect exerted by NO [26]. If NO was involved in the mechanism of the interaction between the two drugs, the synergy degree between them was expected to decrease with an increased abundance of Hmp proteins. In agreement with this hypothesis, overexpression of *hmp* was found to suppress the synergy between DOC and FZ by a factor of 3 (Fig. 5). This finding supports a model that NO generated during FZ metabolism participates in the inhibition of electron transport chain [27], with the secondary effect of inhibiting the function of efflux pumps which are dependent on the electron transport chain for their activity.

### DOC-FZ synergy is largely independent of NfsA/NfsB-mediated FZ activation

**Fig. 6:**
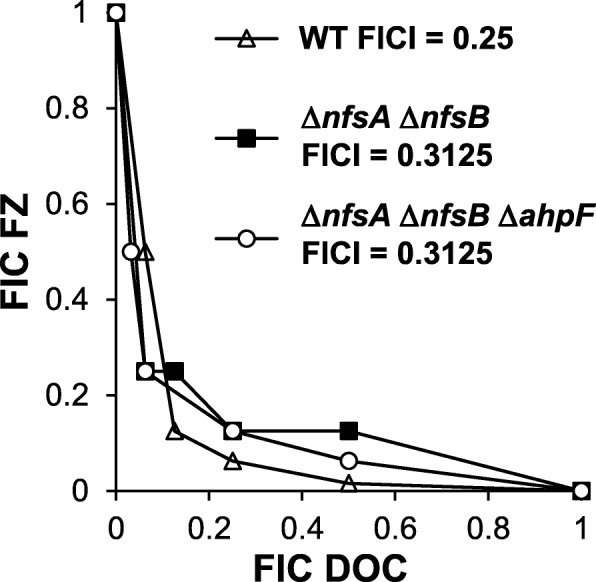
Effect of *nfsA*/*nfsB* deletion on FZ-DOC synergy. Isobologram of FZ-DOC interactions in growth inhibition of the wild type strain (K1508), Δ*nfsA* Δ*nfsB* double and Δ*nfsA* Δ*nfsB* Δ*ahpF* triple mutant. Each data point corresponds to the FIC (ratios of the 50% growth inhibition concentrations in combination *vs.* alone) for FZ (y axis) and DOC (x axis)

It has long been known that nitrofuran drugs need to be activated by nitroreductases NfsA and NfsB to exert its antibacterial activity [13, 14]. As a result, the FZ activity and DOC-FZ synergy may depend on the activity of NfsA and NfsB enzymes. To investigate the role of these two enzymes in the DOC-FZ synergy, we examined the interaction between DOC and FZ in the Δ*nfsA* Δ*nfsB E. coli* strain lacking both of these enzymes. In agreement with the FZ activation role of NfsA/NfsB, disruption of these two genes led to an increase in the MIC causing 50% growth inhibition by a factor of 8 [12]. Nonetheless, the synergy between DOC and FZ still remained significant in the Δ*nfsA* Δ*nfsB* genetic background, with the FICI at 50% growth inhibition as low as 0.3125 (Fig. 6); this FICI value is only slightly higher than that of the wild type strain (0.25). We recently identified a third FZ-activating enzyme in *E. coli*, AhpF, which contributes to this prodrug activation in the Δ*nfsA* Δ*nfsB* genetic background [12]. Nevertheless, FZ was still effective against the Δ*nfsA* Δ*nfsB* Δ*ahpF* mutant with the MIC 50% being 10-fold increased over the wild-type parent, suggesting the presence of additional 5-nitrofuran-activating enzymes in this organism and/or activation-independent mechanisms of action [12]. Here we analyzed the DOC-FZ synergy in the triple Δ*nfsA* Δ*nfsB* Δ*ahpF* mutant and showed that the FICI value was close to that of the wild-type parent and double mutant (Fig. 6). In other words, the contribution of NfsA/NfsB- and AhpF-mediated activation of FZ to the DOC-FZ synergy is very minimal. These findings indicate the role(s) of yet unexplored mechanisms of FZ action or activation in its interaction with DOC.

## Discussion

Capitalization on drug combinations is one of the promising approaches to design novel therapies that will allow application of antimicrobials which have heretofore been ineffective against Gram-negative bacteria at concentrations that are acceptable for medical treatments. The synergistic interaction between DOC and FZ or other nitrofurans against a range of enterobacteria is of this kind. Decrease in growth inhibitory concentrations of nitrofurans when combined with DOC, demonstrated here, is desirable because the lowered concentration has a potential to remove the reported nitrofuran mutagenic and carcinogenic side-effects [15–18]. With respect to DOC and other bile salts, Gram-negative bacteria, such as *E. coli* and *Salmonella enterica*, have evolved high resistance to them using various mechanisms, such as multi-drug efflux pumps, a highly impermeable outer membrane, DNA damage repair machineries, the MqsR/MqsA toxin-antitoxin system and employment of multiple stress responses [9, 28–31]; inclusion of an active agent, such as FZ or other 5-nitrofurans, could reintroduce DOC in the battle against such formidable pathogens. These findings bring about two potential applications.

Firstly, DOC-nitrofuran combinations could be developed for topical applications, such as wound and burn dressings. In 2015, ATX-101 in which deoxycholic acid is the active ingredient was approved by the Food and Drug Administrations for reduction of submental fat at a subcutaneous injection dose as high as 10 mg/mL and a volume of up to 10 mL [32]. This concentration is much higher than that of DOC (2.5 mg/mL) required for observing the synergy between DOC and nitrofurans, indicating that DOC concentrations of less than 10 mg/mL could be used in the combination without a concern about the serious toxicity. In addition to its antibacterial action, one could capitalize on the hydrogel-forming capability of DOC for transdermal drug delivery in DOC-nitrofuran combination. Such uses of DOC have been described in the rat model [33, 34]; no irritant effects on rat skins upon DOC-hydrogel application were observed in the histology studies [34].

Secondly, DOC and other bile salts are inherently present at 2–10 mM concentration range along the gastrointestinal tract, depending on nutritional state and microbiome composition [25, 35]. The efficacy of any drug used to treat intestinal infections would depend on the physicochemical properties of the local environment in which interaction with bile salts is one important factor. For instance, it has been reported that rifaximin, an RNA synthesis inhibitor, was more efficient in treating diarrhea-producing *E. coli* in the intestine than in the colon, due to the difference in the bile salt concentration [36]. We now provide evidence that FZ, an antibiotic prescribed for bacterial diarrhea [11, 37], acts synergistically with DOC in inhibiting the growth of enterobacteria, reducing the MIC of DOC from > 48 mM to 6 mM, the latter within the range of bile salt concentrations in the intestine. It is possible that the synergy in situ may contribute to the treatment. Co-administration of FZ and DOC provides a promising tool to treat bacterial diarrhea, especially for patients with conditions such as malnourishment or disorders in enterohepatic circulation and intestinal absorption, all of which may result in low levels of intestinal bile salts [4]. It should be noted that DOC alone does not represent the intestinal bile salts mixture and therefore application of DOC together with FZ may be necessary to enhance the synergy. LaRusso et al. [38] demonstrated that oral administration of DOC at 750 mg/day in healthy men did not result in any significant side effects even after 2 weeks of application, highlighting the possibility for oral uptake of DOC-FZ combination for bacterial diarrhea.

We have provided insights into the underlying mechanism of the synergy between DOC and FZ in their antibacterial action against *E. coli* as a model Gram-negative bacterium. We showed that disruption of *tolC* or *acrA* gene caused a considerable decrease in the synergy between DOC and FZ in the corresponding mutants. The TolC protein, whose removal disrupts the synergy more strikingly, appears to be the key determinant of synergy.

The observed difference in susceptibility to DOC/FZ combination between Δ*tolC* and Δ*acrA* mutants is in agreement with the fact that the TolC protein is shared by at least seven multidrug efflux pumps, while AcrA protein acts as the periplasmic connecting bridge for only two [39]. Thus, deletion of *tolC* gene is expected to give rise to a more pronounced effect on the loss of efflux activities than deletion of *acrA* gene.

Of great interest is how FZ could influence the activity of efflux pumps. The observed impairment of the DOC-FZ synergy by disruption of genes involved in multiple efflux pumps points to a common mechanism that could affect a wide range of efflux pumps simultaneously, such as proton motive force. It has been suggested that nitrofuran compounds during reductive activation might generate NO which subsequently inhibits the electron transport chain (ETC), diminishing the proton motive force across the cytoplasmic membrane [19, 20, 27] thereby de-energizing multiple efflux pumps and impairing the expulsion of toxic compounds. NO generation from nitrofurans in bacterial cells is, however, speculative, due to the detection limit of the used methods or rapid conversion of NO into other compounds [19, 20]. In the present work, we provide evidence for contribution of NO in the interaction between DOC and FZ via the observation that overexpression of NO-detoxifying enzyme Hmp decreased the synergistic interaction between the two agents. Since some DOC-FZ synergy was still retained after NO-detoxification, other mechanisms, including direct inhibition of the ETC by activated FZ, may be involved in the efflux pump inhibition. Further experiments are warranted to examine the effect of FZ on the electron transport chain via changes in the two components of the proton motive force using various probes (*e.g.* tetramethyl rhodamine methyl ester for membrane electric potential and pHluorin for ΔpH) or by monitoring cellular O_2_ uptake [40].

Notably, we showed that the DOC-FZ synergy does not depend on the presence of two major 5-nitrofuran-activating *E. coli* nitroreductases NfsA and NfsB and a minor activating enzyme AhpF. This finding raises interesting questions about activation and action of nitrofurans. The retention of synergy in the absence of NfsA, NfsB and AhpF implies that the inhibitory effect on the TolC-AcrAB efflux pump via NO is retained and, therefore, FZ likely undergoes reductive activation by alternative enzymes.

## Conclusions

The current study reports the synergy between FZ and DOC in inhibiting and/or killing several enterobacterial species at concentrations that are demonstrated to be non-toxic in animal and human trials, and within the range of intestinal bile salts concentrations. We provide genetic evidence that the efflux pumps play a major role in the FZ-DOC synergy, suggesting that the mechanism of synergy may be a 5-nitrofuran-mediated increase in accumulation of DOC inside the cell. In support of this model, we show that the key enzyme which detoxifies NO, an FZ-activation product that inhibits ETC, also impairs the FZ-DOC synergy.

## Methods

### Bacterial strains, growth conditions and antibiotics

All bacterial strains and plasmids used in this study were described in Table 1 and Table 2. The introduction of the *kan*^*R*^ gene deletion mutations into the wild type strain K1508 from the corresponding Keio collection *E. coli* K12 knock-out strains [45] was performed using phage P1 transduction, according to the standard procedures [46]. To eliminate potential polar effects on downstream genes in the operon, the FRT-flanked *kan*^*R*^ cassette was then removed using FLP-mediated recombination as previously described [47]. Plasmids derived from the pCA24N bearing the gene of interest were purified from *E. coli* strains of the ASKA collection containing ORF expression constructs derived from this organism [44] using the ChargeSwitch-Pro Plasmid Miniprep Kit (Thermo Fisher Scientific). The plasmid DNA was then chemically transformed into specific *E. coli* strains for further work [48]. Expression from the pCA24N vector is driven from a T5-*lac* chimeric promoter. In the case of membrane protein expression (TolC and AcrA), the basal expression from uninduced promoter was used in complementation experiments to avoid toxicity of membrane protein overexpression due to the Sec system saturation, whereas expression of Hmp (a cytosolic NO-detoxifying protein) was induced by 1 mM IPTG.

**Table 1:** Bacterial strains used in this study

Name	Genotype or description	Source
*Escherichia coli* O157 isolate *ERL034336*	Human isolate	Dr. Ann Midwinter, School of Veterinary Sciences, Massey University, Palmerston North, New Zealand
*Escherichia coli* UPEC P50 isolate	Isolate from a canine urinary tract infection	[41]
*Salmonella enterica* LT2	Type strain, *S. enterica* subsp. *enterica,* serovar Typhimurium	ATCC® 43971™
*Citrobacter gillenii* PMR001	Isolate from a municipal sewage processing (water purification) plant, Palmerston North, New Zealand (classified by complete 16S rRNA sequencing, 99% identity over 1405 nt to the 16S rRNA sequence of *Citrobacter gillenii* ATCC 51117).	This study
*Klebsiella pneumoniae* PMR001	Isolate from a municipal sewage processing (water purification) plant, Palmerston North, New Zealand (classified by complete 16S rRNA sequencing; 99% identity over 1404 nt to the 16S rRNA sequence of *Klebsiella pneumoniae* strain ATCC 13883).	This study
*Escherichia coli* K12 laboratory strains		
K1508	MC4100 [*F*^−^ *araD*^*−*^ Δ*lac* U169 *relA*^*−*^ *thiA rpsL* (Str^R^)] Δ*lamB106*	[42]
K2403	K1508 Δ*tolC*	This study
K2424	K1508 Δ*acrA*	This study
K2425	K1508 Δ*acrA* pCA24N*::acrA* Δ*gfp*	This study
K2426	K1508 Δ*tolC* pCA24N*::tolC* Δ*gfp*	This study
K2483	K1508 Δ*nfsA* Δ*nfsB*	This study
K2505	K1508 Δ*nfsA* Δ*nfsB* Δ*ahpF*	This study
K2524	K1508 pUC118 (Amp^R^)	This study

**Table 2:** List of plasmids used in this study

Name	Genotype or description	Source
pCP20	Amp^R^, Cm^R^, FLP^+^, 8 cI857^+^, 8 *p*_R_ Rep^ts^ For removal of an *frt*-flanked *kan* marker from *E. coli* K12 strains by FLP-mediated site-specific recombination	[43]
pUC118	Amp^R^, f1 *ori*, *P*_*lacUV5*_, *lacZα*	Creative Biogene, Shirley, NY, USA
pCA24N-*tolC*	Cm^R^; *lacI*^q^, pCA24N P_T5-lac_::*tolC* Δ*gfp*	[44]
pCA24N-*acrA*	Cm^R^; *lacI*^q^, pCA24N P_T5-lac_::*acrA* Δ*gfp*	[44]
pCA24N-*hmp*	Cm^R^; *lacI*^q^, pCA24N P_T5-lac_::*hmp* Δ*gfp*	[44]

Bacterial culture was grown in 2xYT medium (BD Difco) at 37 °C with shaking at 200 rpm. For preparation of exponential phase cells, fresh overnight culture was 100-fold diluted and incubated to reach the OD_600nm_ of about 0.1–0.3. This cell suspension was then diluted to the desirable concentration depending on specific purposes. Sodium Deoxycholate was a kind gift from New Zealand Pharmaceuticals Ltd. Antibiotics used in this study were purchased from GoldBio. CM4 was purchased from Enamine (catalog number Z49681516).

### Checkerboard assay

The checkerboard assay for DOC and FZ was performed on the Corning 384-well microtiter plate with the concentration of DOC ranging from 20,000 μg/mL to 0 μg/mL and the concentration of FZ ranging from 10 μg/mL to 0 μg/mL, prepared by 2-fold serial dilution. The concentration range could be adjusted depending on the sensitivity of different bacterial strains and the types of nitrofurans to cover at least 2 × MIC to 0.06 × MIC of each drug. Each well contained the starting inoculum of approximately 10^6^ CFU/mL, 2% DMSO and the predefined concentration of each drug in the total volume of 50 μL. The wells containing no drugs and 10 μg/mL tetracycline were used as negative controls and positive controls, respectively. After dispensing the reagents, the plate was pulse centrifuged at 1000×g to eliminate any bubbles. The plate was then incubated at 30 °C and the OD_600nm_ of the sample was monitored for every 1 h within 24 h using Multiskan™ GO Microplate Spectrophotometer (Thermo Scientific). Each combination was performed in triplicate. The mean growth inhibition of the triplicate experiments with the cut-off value of 90% at the time point 24 h was used to define the MIC of the drug used alone or in combination [49]. The fractional inhibitory concentration index (FICI) for the two drugs was calculated as follows:

$$ \mathrm{FICI}=\frac{MI{C}_{DOCcom}}{MI{C}_{DOCalone}}+\frac{MI{C}_{FZcom}}{MI{C}_{FZalone}} $$

MIC_DOCcom_ and MIC_FZcom_: MIC of DOC and FZ when tested in combination.

MIC_DOCalone_ and MIC_FZalone_: MIC of DOC and FZ when tested individually.

The interaction between two drugs was interpreted as synergistic if FICI was ≤0.5, indifferent if it was > 0.5 and ≤ 4, and antagonistic if it was > 4 [50]. The 50% growth inhibition was used as the cut-off value to calculate FICI in some cases when stated.

### Time-kill assay

Exponential phase bacterial culture at about 10^6^ CFU/mL was prepared in the final volume of 10 mL containing 2% DMSO plus DOC at 2500 μg/mL alone or FZ at 0.5 × MIC μg/mL alone or both drugs. The treatments containing no drug were used as negative controls. The samples were incubated at 30 °C with shaking at 200 rpm. At the time points of 0 h, 2 h, 4 h, 6 h, 8 h and 24 h, 500 μL were taken from each treatment and centrifuged at 10000×g for 15 min before being re-suspended in 100 μL maximum recovery diluent (0.1% peptone, 0.85% NaCl). 10 μL of serial dilutions was plated on 2xYT agar followed by overnight incubation at 37 °C to determine the cell count. Each treatment was performed in triplicate. The antimicrobial interaction was interpreted as synergistic if the combinatorial treatment caused a killing efficiency ≥2 log higher than the most active agent [51].

## Supplementary information

**Additional file 1:** **Figure S1.** Structural formulae of nitrofurans and sodium deoxycholate. **Figure S2.** FZ interaction with DOC in growth inhibition of *E. coli* strain O157 and canine uropathogenic *E. coli* P50. **Figure S3.** Interactions of three nitrofurans (NIT, NFZ and CM4) with DOC in growth inhibition of *Citrobacter gillenii* PMR001. **Figure S4.** Interactions of three nitrofurans (NIT, NFZ and CM4) with DOC in growth inhibition of *Salmonella enterica* sv. Typhimurium LT2. **Figure S5.** Interactions of two nitrofurans (NIT and NFZ) with DOC in growth inhibition of *Klebsiella pneumoniae* PMR001.

## Data Availability

All data generated or analyzed during this study are included in this published article and its supplementary information file. The 16S rDNA sequence of the isolates *Citrobacter gillennii* PMR001 and *Klebsiella pneumoniae* PMR001 has been deposited at GenBank under the accession numbers MN515064 and MN515061, respectively.

## References

[1] O’Neill J. Antimicrobial resistance: tackling a crisis for the health and wealth of nations. Rev Antimicrob Resist. 2014; https://amr-review.org/Publications.html.

[2] Bollenbach T (2015). Antimicrobial interactions: mechanisms and implications for drug discovery and resistance evolution. Curr Opin Microbiol.

[3] Taneja N, Kaur H (2016). Insights into newer antimicrobial agents against gram-negative bacteria. Microbiol Insights.

[4] Begley M, Gahan CG, Hill C (2005). The interaction between bacteria and bile. FEMS Microbiol Rev.

[5] Merritt ME, Donaldson JR (2009). Effect of bile salts on the DNA and membrane integrity of enteric bacteria. J Med Microbiol.

[6] Cremers CM, Knoefler D, Vitvitsky V, Banerjee R, Jakob U (2014). Bile salts act as effective protein-unfolding agents and instigators of disulfide stress in vivo. P Natl Acad Sci USA.

[7] Nishino K, Yamaguchi A (2001). Analysis of a complete library of putative drug transporter genes in *Escherichia coli*. J Bacteriol.

[8] Paul S, Alegre KO, Holdsworth SR, Rice M, Brown JA, McVeigh P (2014). A single-component multidrug transporter of the major facilitator superfamily is part of a network that protects *Escherichia coli* from bile salt stress. Mol Microbiol.

[9] Sistrunk JR, Nickerson KP, Chanin RB, Rasko DA, Faherty CS (2016). Survival of the fittest: how bacterial pathogens utilize bile to enhance infection. Clin Microbiol Rev.

[10] Chamberlain RE (1976). Chemotherapeutic properties of prominent nitrofurans. J Antimicrob Chemother.

[11] Vass M, Hruska K, Franek M (2008). Nitrofuran antibiotics: a review on the application, prohibition and residual analysis. Vet Med-Czech.

[12] Le VVH, Davies I, Moon CD, Wheeler D, Biggs PJ, Rakonjac J. Novel 5-nitrofuran-activating reductase in *Escherichia coli*. Antimicrob Agents Chemother. 2019. 10.1128/AAC.00868-19. 10.1128/AAC.00868-19 PMC6811407 31481448

[13] Whiteway J, Koziarz P, Veall J, Sandhu N, Kumar P, Hoecher B (1998). Oxygen-insensitive nitroreductases: analysis of the roles of *nfsA* and *nfsB* in development of resistance to 5-nitrofuran derivatives in *Escherichia coli*. J Bacteriol.

[14] Sandegren L, Lindqvist A, Kahlmeter G, Andersson DI (2008). Nitrofurantoin resistance mechanism and fitness cost in *Escherichia coli*. J Antimicrob Chemother.

[15] McCalla DR, Hahn FE (1979). Nitrofurans. Mechanism of Action of Antibacterial Agents; Antibiotics.

[16] McOsker CC, Fitzpatrick PM (1994). Nitrofurantoin: mechanism of action and implications for resistance development in common uropathogens. J Antimicrob Chemother.

[17] Bertenyi KK, Lambert IB (1996). The mutational specificity of furazolidone in the *lacI* gene of *Escherichia coli*. Mutat Res.

[18] Ona KR, Courcelle CT, Courcelle J (2009). Nucleotide excision repair is a predominant mechanism for processing nitrofurazone-induced DNA damage in *Escherichia coli*. J Bacteriol.

[19] Kumar M, Adhikari S, Hurdle JG (2014). Action of nitroheterocyclic drugs against *Clostridium difficile*. Int J Antimicrob Agents.

[20] Vumma R, Bang CS, Kruse R, Johansson K, Persson K (2016). Antibacterial effects of nitric oxide on uropathogenic *Escherichia coli* during bladder epithelial cell colonization--a comparison with nitrofurantoin. J Antibiot (Tokyo).

[21] Roldan MD, Perez-Reinado E, Castillo F, Moreno-Vivian C (2008). Reduction of polynitroaromatic compounds: the bacterial nitroreductases. FEMS Microbiol Rev.

[22] Hall BS, Bot C, Wilkinson SR (2011). Nifurtimox activation by trypanosomal type I nitroreductases generates cytotoxic nitrile metabolites. J Biol Chem.

[23] Voak AA, Gobalakrishnapillai V, Seifert K, Balczo E, Hu L, Hall BS (2013). An essential type I nitroreductase from *Leishmania major* can be used to activate leishmanicidal prodrugs. J Biol Chem.

[24] Akiva E, Copp JN, Tokuriki N, Babbitt PC (2017). Evolutionary and molecular foundations of multiple contemporary functions of the nitroreductase superfamily. Proc Natl Acad Sci U S A.

[25] Holm R, Mullertz A, Mu HL (2013). Bile salts and their importance for drug absorption. Int J Pharm.

[26] Forrester MT, Foster MW (2012). Protection from nitrosative stress: a central role for microbial flavohemoglobin. Free Radic Biol Med.

[27] McCollister BD, Hoffman M, Husain M, Vazquez-Torres A (2011). Nitric oxide protects Bacteria from aminoglycosides by blocking the energy-dependent phases of drug uptake. Antimicrob Agents Chemother.

[28] Prieto AI, Ramos-Morales F, Casadesus J (2006). Repair of DNA damage induced by bile salts in *Salmonella enterica*. Genetics.

[29] Kwan BW, Lord DM, Peti W, Page R, Benedik MJ, Wood TK (2015). The MqsR/MqsA toxin/antitoxin system protects *Escherichia coli* during bile acid stress. Environ Microbiol.

[30] Urdaneta V, Casadesus J (2017). Interactions between bacteria and bile salts in the gastrointestinal and hepatobiliary tracts. Front Med.

[31] Urdaneta V, Casadesus J (2018). Adaptation of *Salmonella enterica* to bile: essential role of AcrAB-mediated efflux. Environ Microbiol.

[32] Dunican KC, Patel DK (2016). Deoxycholic acid (ATX-101) for reduction of submental fat. Ann Pharmacother.

[33] Valenta C, Nowack E, Bernkop-Schnurch A (1999). Deoxycholate-hydrogels: novel drug carrier systems for topical use. Int J Pharm.

[34] Senyigit T, Tekmen I, Sonmez U, Santi P, Ozer O (2011). Deoxycholate hydrogels of betamethasone-17-valerate intended for topical use: in vitro and in vivo evaluation. Int J Pharm.

[35] Enright EF, Griffin BT, Gahan CGM, Joyce SA (2018). Microbiome-mediated bile acid modification: role in intestinal drug absorption and metabolism. Pharmacol Res.

[36] Darkoh C, Lichtenberger LM, Ajami N, Dial EJ, Jiang ZD, DuPont HL (2010). Bile acids improve the antimicrobial effect of rifaximin. Antimicrob Agents Chemother.

[37] Martinez-Puchol S, Gomes C, Pons MJ, Ruiz-Roldan L, Torrents de la Pena A, Ochoa TJ (2015). Development and analysis of furazolidone-resistant *Escherichia coli* mutants. APMIS.

[38] LaRusso NF, Szczepanik PA, Hofmann AF (1977). Effect of deoxycholic acid ingestion on bile acid metabolism and biliary lipid secretion in normal subjects. Gastroenterology.

[39] Anes J, McCusker MP, Fanning S, Martins M (2015). The ins and outs of RND efflux pumps in *Escherichia coli*. Front Microbiol.

[40] Chen MT, Lo CJ (2016). Using biophysics to monitor the essential proton motive force in bacteria. Adv Exp Med Biol.

[41] Le VVH, Bruce I, Biggs PJ, Rakonjac J (2019). Draft genome sequence of a canine uropathogenic *Escherichia coli* strain isolated in New Zealand. Microbiol Resour Announc.

[42] Spagnuolo J, Opalka N, Wen WX, Gagic D, Chabaud E, Bellini P (2010). Identification of the gate regions in the primary structure of the secretin pIV. Mol Microbiol.

[43] Cherepanov PP, Wackernagel W (1995). Gene disruption in *Escherichia coli*: TcR and KmR cassettes with the option of Flp-catalyzed excision of the antibiotic-resistance determinant. Gene.

[44] Kitagawa M, Ara T, Arifuzzaman M, Ioka-Nakamichi T, Inamoto E, Toyonaga H (2005). Complete set of ORF clones of *Escherichia coli* ASKA library (a complete set of *E. coli* K-12 ORF archive): unique resources for biological research. DNA Res.

[45] Baba T, Ara T, Hasegawa M, Takai Y, Okumura Y, Baba M (2006). Construction of *Escherichia coli* K-12 in-frame, single-gene knockout mutants: the Keio collection. Mol Syst Biol.

[46] Thomason LC, Costantino N, Court DL (2007). *E. coli* genome manipulation by P1 transduction. Curr Protoc Mol Biol.

[47] Datsenko KA, Wanner BL (2000). One-step inactivation of chromosomal genes in *Escherichia coli* K-12 using PCR products. Proc Natl Acad Sci U S A.

[48] Green R, Rogers EJ (2013). Transformation of chemically competent *E. coli*. Methods Enzymol.

[49] Campbell J (2010). High-throughput assessment of bacterial growth inhibition by optical density measurements. Curr Protoc Chem Biol.

[50] Odds FC (2003). Synergy, antagonism, and what the chequerboard puts between them. J Antimicrob Chemoth.

[51] Doern CD (2014). When does 2 plus 2 equal 5? A review of antimicrobial synergy testing. J Clin Microbiol.

