# Supplementary material for: In vitro synergy between sodium deoxycholate and furazolidone against enterobacteria
Source: BMC Microbiol. 2020 Jan 6;20:5. doi: 10.1186/s12866-019-1668-3 (PMC6945529; doi:10.1186/s12866-019-1668-3)
Supplement: Supplementary file 1 — Additional file 1: Figure S1. Structural formulae of nitrofurans and sodium deoxycholate. Figure S2. FZ interaction with DOC in growth inhibition of E. coli strain O157 and canine uropathogenic E. coli P50. Figure S3. Interactions of three nitrofurans (NIT, NFZ and CM4) with DOC in growth inhibition of Citrobacter gillenii PMR001. Figure S4. Interactions of three nitrofurans (NIT, NFZ and CM4) with DOC in growth inhibition of Salmonella enterica sv. Typhimurium LT2. Figure S5. Interactions of two nitrofurans (NIT and NFZ) with DOC in growth inhibition of Klebsiella pneumoniae PMR001. [file 12866_2019_1668_MOESM1_ESM.pdf]

In vitro Synergy between Sodium Deoxycholate and Furazolidone against Enterobacteria

Vuong Van Hung Le[a], Catrina Olivera[a], Julian Spagnuolo[a1], Ieuan Davies[b] and Jasna Rakonjac[a]*

[a]School of Fundamental Sciences, Massey University, Palmerston North, New Zealand

[b]New Zealand Pharmaceuticals Ltd., Palmerston North, New Zealand

[1]**Present address:** Department of Biomedicine, University Hospital Basel, 4031 Basel, Switzerland

***Corresponding author:** Jasna Rakonjac, School of Fundamental Sciences, Massey University, Palmerston North, New Zealand

The Content of Supplementary Data:

- **Figure S1:** Structural formulae of nitrofurans and sodium deoxycholate
- **Figure S2:** FZ interaction with DOC in growth inhibition of *E. coli* strain O157 (A) and canine uropathogenic *E. coli* P50 (B)
- **Figure S3:** Interactions of three nitrofurans (NIT, NFZ and CM4) with DOC in growth inhibition of *Citrobacter gillenii* PMR001
- **Figure S4:** Interactions of three nitrofurans (NIT, NFZ and CM4) with DOC in growth inhibition of *Salmonella enterica* sv. Typhimurium LT2
- **Figure S5:** Interactions of two nitrofurans (NIT and NFZ) with DOC in growth inhibition of *Klebsiella pneumoniae* PMR001

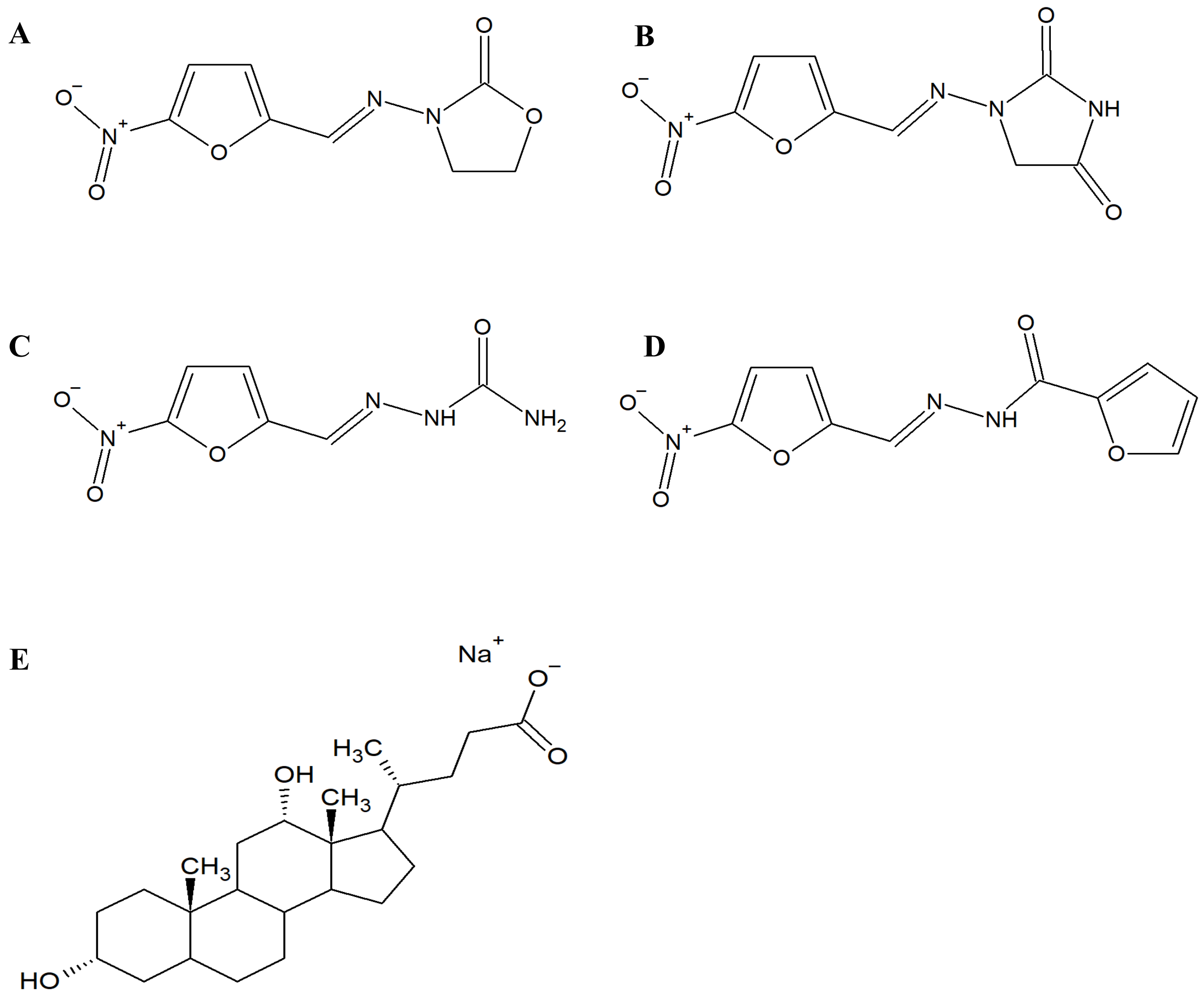

Figure S1: Structural formulae of nitrofurans and sodium deoxycholate (E). A) Furazolidone (FZ); B) Nitrofurantoin (NIT); C) Nitrofurazone (NFZ). D) CM4, Pubchem ID AC1LGLMG (no CAS number). Chemical name: N'-[(5-nitrofuran-2-yl)methylidene]furan-2-carbohydrazide or N-[(5-nitrofuran-2-yl)methylideneamino]furan-2-carboxamide.

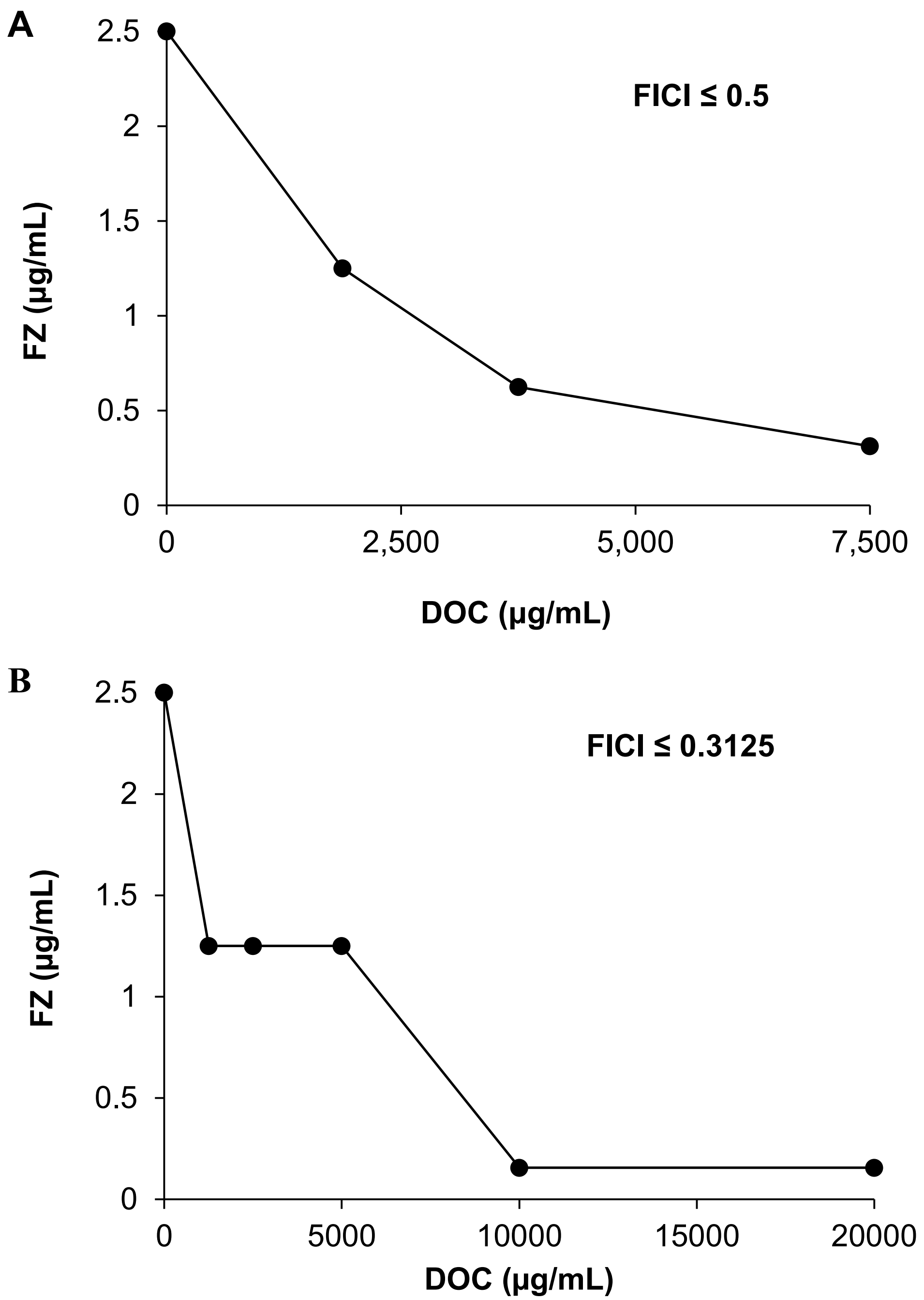

Figure S2: FZ interaction with DOC in growth inhibition of *E. coli* strain O157 (A) and canine uropathogenic *E. coli* P50 (B). Graphs (isobolograms) are obtained using a checkerboard analysis at multiple concentration of molecules. Each data point represents the minimum molecule concentrations alone or in combination causing 90% inhibition to bacterial growth.

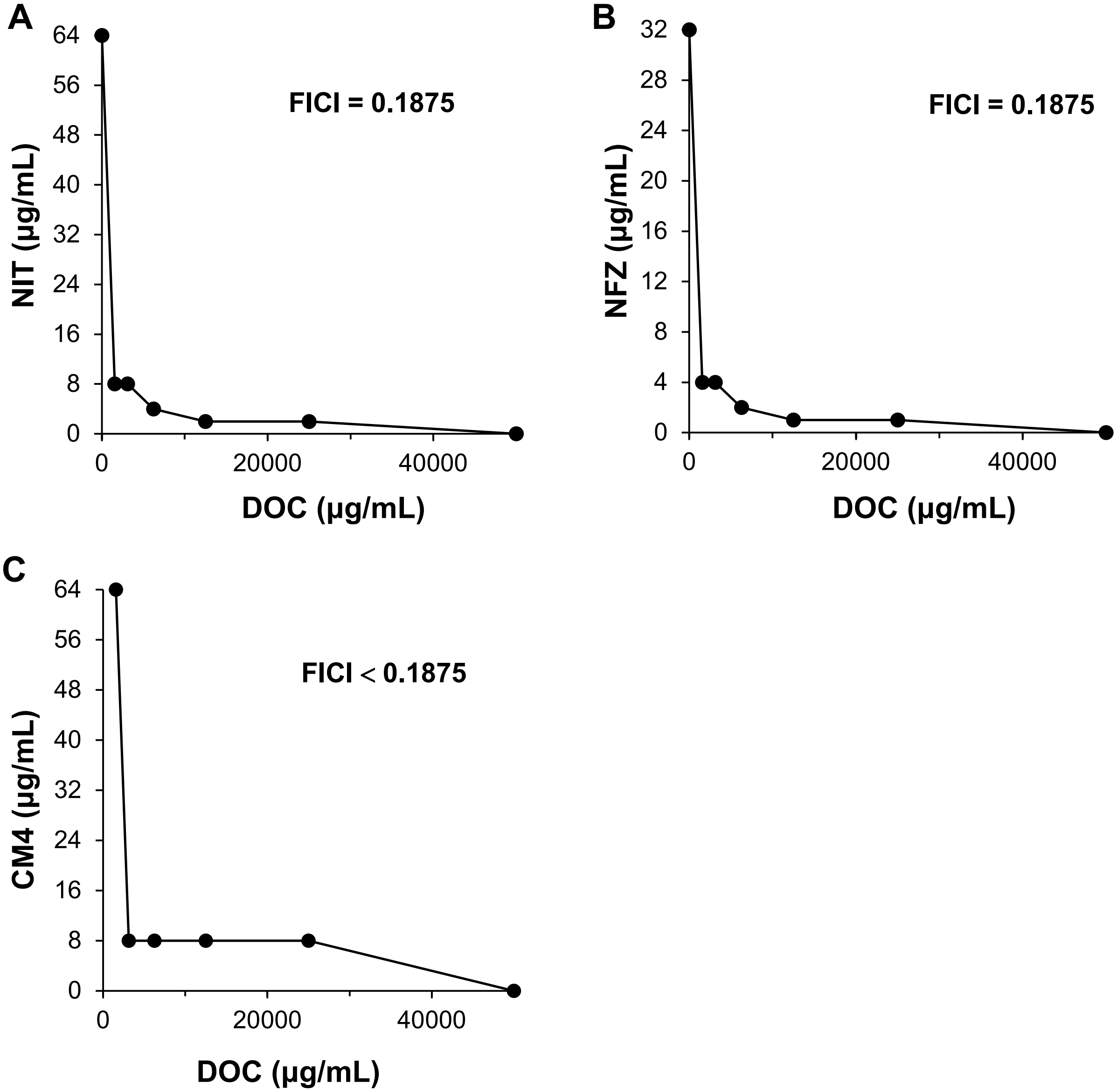

Figure S3: Interactions of three nitrofurans (NIT, NFZ and CM4) with DOC in growth inhibition of *Citrobacter gillenii* PMR001. Graphs (isobolograms) are obtained using a checkerboard analysis at multiple concentration of molecules. Each data point represents the minimum molecule concentrations alone or in combination causing 90% inhibition to bacterial growth.

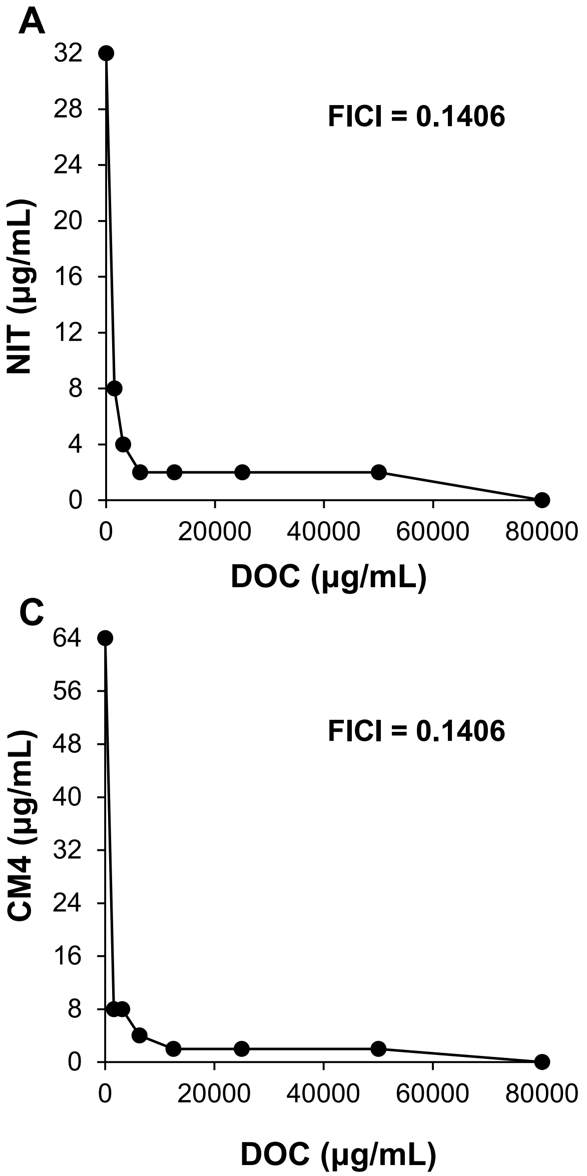

Figure S4: Interactions of three nitrofurans (NIT, NFZ and CM4) with DOC in growth inhibition of *Salmonella enterica* sv. Typhimurium LT2. Graphs (isobolograms) are obtained using a checkerboard analysis at multiple concentration of molecules. Each data point represents the minimum molecule concentrations alone or in combination causing 90% inhibition to bacterial growth.

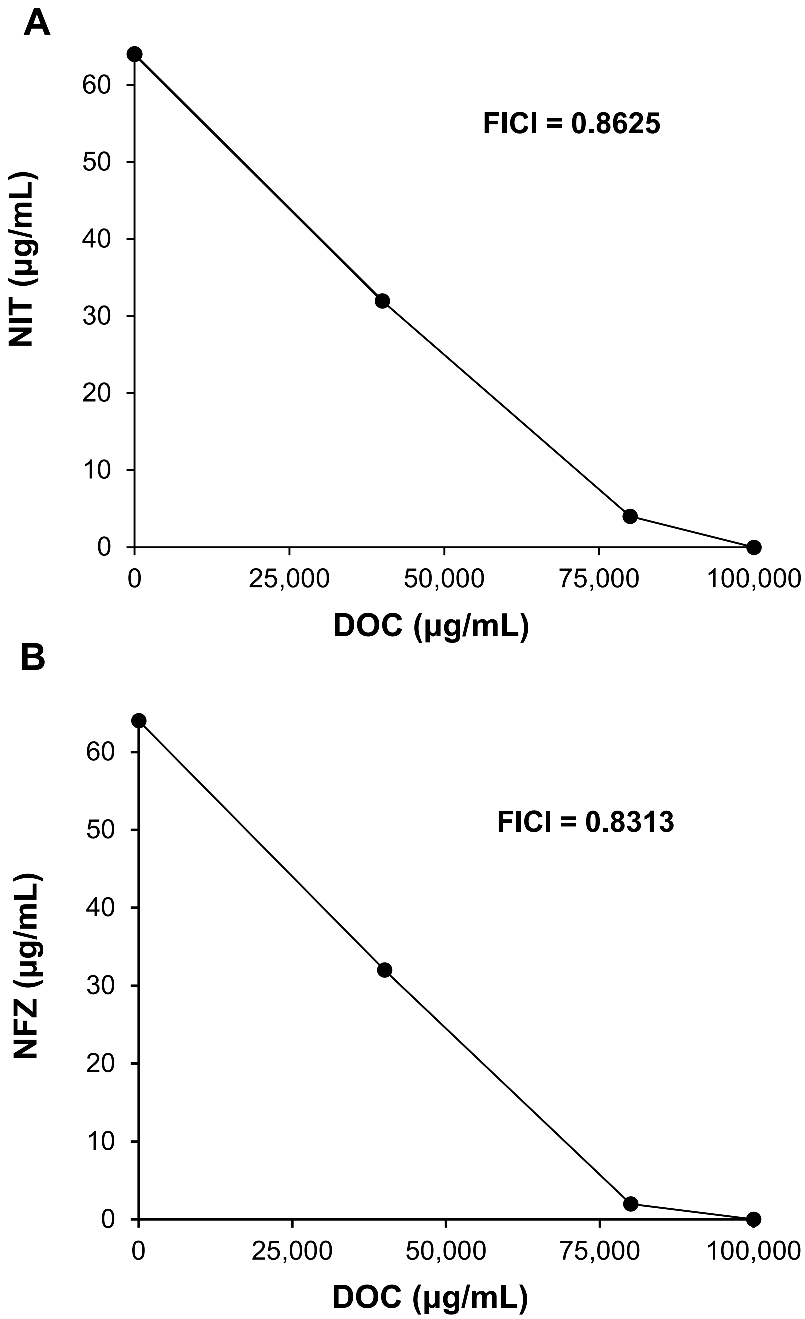

Figure S5: Interactions of two nitrofurans (NIT and NFZ) with DOC in growth inhibition of *Klebsiella pneumoniae* PMR001. Graphs (isobolograms) are obtained using a checkerboard analysis at multiple concentration of molecules. Each data point represents the minimum molecule concentrations alone or in combination causing 90% inhibition to bacterial growth.
